# Practical Notes on Diagnosis and Treatment

**Published:** 1910-12-24

**Authors:** 


### Practical Notes on Diagnosis and Treatment.

**Genital Hæmorrhage in Babies.**

This occurs but rarely in male infants, but several cases have been reported of hæmorrhage from the vagina in babies. The explanation of the bleeding is not quite certain, but it seems to be dependent upon the presence of pelvic congestion, which has resulted from the sudden obliteration of the umbilical arteries; it is generally recovered from.—*Mr. A. H. Tubby.*

**Adrenalin for Shock.**

To be really efficacious, adrenalin in normal saline solution should be introduced directly into the veins, since, if used hypodermically or by the rectum its first effect is to blanch the surrounding tissues, with the result that it does not at once pass into the circulation, and its general effect is greatly interfered with. I have frequently used a drachm of the solution to the pint of saline in carrying out an intravenous injection with the best results.—*Mr. Lawrie McGavin.*

**Phosphaturia.**

It is only the earthy phosphates of calcium and magnesium that can form a deposit. Such a deposit may occur (1) on boiling the urine, (2) as an iridescent pellicle on the surface of the urine when it has been passed, (3) in the bladder, so that the last portion of the urine is milky; this is often mistaken for spermatorrhœa, and the patient becomes needlessly depressed and falls an easy prey to quacks. Phosphaturia is very common in hyperchlorhydria, as if there is an excessive secretion of hydrochloric acid in the gastric juice there will be less acid at the disposal of the urine. I always look for hyperchlorhydria to confirm my diagnosis.—*Dr. Langdon Brown.*

**B. Coli in the Urethra.**

That the bacillus coli is a common denizen of the urethra has been pointed out by Melchior, who found it present in the meatus urinarius in 50 per cent. of all cases examined. Hence it is conceivable that, in spite of every care, organisms may be pushed into the bladder by instrumentation.—*Dr. Batty Shaw.*

**Pneumococcal Endocarditis.**

Of cardiac affections due to the pneumococcus, endocarditis is by far the most serious; it is either associated with pneumonia or is met with as an independent disease; at least we cannot always find a primary source. With anatomcial features of its own, it is perhaps more often dextral than the endocardial lesion of any other organism, and in a high degree it shares with the streptococcus the property of malignancy. Very few cases recover, and many are overlooked clinically, as the cardiac features of the case may be completely masked in a profound toxæmia.—*Professor Wm. Osler.*

**Retro-pharyngeal Abscess.**

This affection may be met with in two forms, acute or tuberculous. The former is seen in small children, and probably starts in the lymphatic glands, which lie in front of the pre-vertebral aponeurosis about the level of the second cervical vertebra. The tuberculous variety may start in the same glands or be connected with disease of the vertebra. If time and circumstances permit, both varieties are better opened from the side of the neck, though it must be confessed that most cases of the acute abscess do well when opened through the mouth; but this should never be done if the abscess is tuberculous.—*Mr. Aslett Baldwin.*

